# Prophylactic Role of *Averrhoa carambola* (Star Fruit) Extract against Chemically Induced Hepatocellular Carcinoma in Swiss Albino Mice

**DOI:** 10.1155/2014/158936

**Published:** 2014-02-19

**Authors:** Ritu Singh, Jyoti Sharma, P. K. Goyal

**Affiliations:** Radiation & Cancer Biology Laboratory, Department of Zoology, University of Rajasthan, Jaipur 302 004, India

## Abstract

Liver cancer remains one of the severe lethal malignancies worldwide and hepatocellular carcinoma (HCC) is the most common form. The current study was designed to evaluate the prophylactic role of the fruit of *Averrhoa carambola* (star fruit or Kamrak) on diethylnitrosamine- (DENA-) induced (15 mg/kg b.wt.; single i.p. injection) and CCl_4_-promoted (1.6 g/kg b.wt. in corn oil thrice a week for 24 weeks) liver cancer in Swiss albino mice. Administration of ACE was made orally at a dose of 25 mg/kg b.wt/day for 5 consecutive days and it was withdrawn 48 hrs before the first administration of DENA (preinitiational stage). CCl_4_ was given after 2 weeks of DENA administration. A cent percent tumor incidence was noted in carcinogen treated animals while ACE administration resulted in a considerable reduction in tumor incidence, tumor yield, and tumor burden. Further, ACE treatment brings out a significant reduction in lipid peroxidation (*P* < 0.001) along with an elevation in the activities of enzymatic antioxidants (superoxide dismutase, *P* < 0.001, and catalase, *P* < 0.001), nonenzymatic antioxidant (reduced glutathione, *P* < 0.001), and total proteins (*P* < 0.001) when compared to the carcinogen treated control. These results demonstrate that ACE prevents the DENA/CCl_4_ induced adverse physical and biochemical alterations during hepatic carcinogenesis in mice. This study suggests the prophylactic role of *Averrhoa carambola* against hepatocellular carcinoma in mice; therefore, it could be employed for the further screening as a good chemopreventive natural supplement against cancer.

## 1. Introduction

Cancer is a global challenge as this disease remains the second largest cause of death around the world, with some predictions that it will move into the top rank in coming time. Cancer accounts for one out of every eight deaths annually. Increase in life expectancy and adoption of western diet and lifestyles, owing to tobacco abuse and widespread exposure to carcinogens, are some of the major key factors for increasing the burden of cancer in the developing countries like India.

Hepatocellular carcinoma (HCC) is one of the most frequent malignant tumors worldwide and a leading cause of cancer related death killing 5 lacs people annually [1]. HCC has been linked to diverse etiologies including chronic hepatitis B and C viral infection and alcohol exposure [2]. Due to the high tolerance of liver, HCC is seldom detected at the early stage and treatment has a poor prognosis in most of the cases, making it a significant global health problem [3–5]. The recurrence rates of HCC are also very high and long-term survival rate of the patients has not improved much from the past few decades. Surgery, including transplantation resection, is currently the most effective treatment for HCC.

Since the liver is the major site of metabolism of ingested materials, it is more susceptible to carcinogenic insult. DENA (diethylnitrosamine) is a potent carcinogen entering the environment through the food chain [6]. DENA is synthesized endogenously and found in work place, processed meats, tobacco smoke, whiskey, and wide variety of foods, and it is also produced from metabolism of some drugs [7]. In addition, DENA is extensively used as a solvent in fiber industry, softener for copolymer, additive for lubricants, in condensers to increase the dielectric constant, and for the synthesis of 1,1-diethylhydrazine [8]. It is a potent hepatocarcinogen known to cause perturbations in the nuclear enzymes involved in DNA repair or replication [9].

Free radicals and other reactive oxygen species (ROS) are constantly formed in the human body accumulation which causes oxidative damage. Normal cells have evolved defense mechanisms for protection against this oxidative damage by developing multiple antioxidative defenses [10]. If such delicate balance of free radical production and antioxidant defenses goes out of control, it results in the pathology of several human diseases, including cancer, atherosclerosis, malaria, rheumatoid arthritis, and neurodegenerative diseases [11, 12].

Chemoprevention is a pharmacological way of interference in order to arrest or reverse the process of carcinogenesis. Chemopreventive substances are identified on the basis of their antioxidant, antimutagenic, and anti-inflammatory activities capable of arresting proliferation and enhancing apoptosis which are the major criteria for their anticarcinogenic activity. Progress in the area of chemoprevention during the past two decades has been very impressive. Accumulating epidemiological and experimental evidences have revealed the chemopreventive influence of number of naturally occurring compounds and their role in prevention of the diseases [13–17].

Herbal products are gaining progressively attention these days for primary health care owing to less toxicity, better compatibility with the body, and high efficacy against free radical mediated diseases. Many studies have suggested that a healthy diet, especially fruits and vegetables that are rich in natural antioxidants, is efficacious to prevent oxidative stress and thus plays a vital role in cancer prevention [18].

*Averrhoa carambola* L. (Oxalidaceae) is also known as the star fruit tree. Studies have shown that the fruit of *A. carambola* has several medicinal properties and it is rich in antioxidants which act against reactive oxygen species. The ripe star fruit has digestive and biliousness properties. It is also a good source of vitamin C and used to treat headache, vomiting, coughing, hangovers, and eczemas [19, 20]. Furthermore, it is used as an appetite stimulant, diuretic, antidiarrheal, and febrifugal agent. In addition, the extract obtained through the leaves of such planthas been used in the treatment of diabetes [21].

Looking into the pharmacological and medicinal properties of this plant, the present study has been targeted to investigate the possible anticancer potential of *A. carambola* fruit extract against chemical induced hepatocellular carcinoma in mammals.

## 2. Materials and Methods

### 2.1. Chemicals

Diethylnitrosamine (DENA) and carbon tetrachloride (CCl_4_) were purchased from Sigma Chemical Co. (St. Louis, MO, USA). DENA at a dose 15 mg/kg b.wt (single i.p. injection in normal saline) was injected to initiate hepatic carcinogenesis, while CCl_4_ (1.6 g/kg b.wt.) in 1 : 1 dilution with corn oil was given orally to animals by gavage to stimulate liver cell proliferation and regeneration.

### 2.2. Preparation of Plant Extract

Carambola fruits were cleaned, air dried, and grinded into the form of fine powder. The powder was extracted with 90% ethyl alcohol using Soxhlet apparatus and concentrated by evaporating its liquid contents. The required dose for further treatment was prepared by dissolving the extract in DDW.

### 2.3. Animals

Swiss albino mice (3-week old) were taken for the experiment from an inbred colony, and they were provided feed and water *ad libitum*. All studies were carried out in accordance with the guidelines of the Institutional Animal Ethics Committee & INSA, New Delhi.

### 2.4. Dose Selection of ACE

For deciding the optimum dose, experiments were conducted in which Swiss albino mice were divided into different groups and were given orally *Averrhoa carambola* extract (ACE) at the dose of 05, 15, 25, 50, and 75 mg/kg b.wt./day mg/animal/day. Animals from each group were observed for 30 days for any sign of sickness, morbidity, mortality, gait, weight, behavioral alterations, and so forth, and were necropsied on 16th and 31st day. Various doses of ACE were selected (i.e., 15, 25, and 50 mg/kg/b.wt./animal), from the above doses, after evaluation of various biochemical parameters in the liver of mice. Out of these, 25 mg dose was found to be the optimum dose for this experiment.

### 2.5. Chemopreventive Activity of ACE

Animals for this experiment were divided into the following groups.

*Group I: Negative Control (Vehicle Treated Normal Mice).* In this group, animals were given single i.p. injection of normal saline and later administered with corn oil by oral gavage, three times in a week for the entire experimental period, that is, for 24 weeks.

*Group II: Positive Control (Carcinogen Treated).* The animals in this group were given DENA in normal saline. After 2 weeks of DENA administration, CCl_4_ was given 3 times in a week until the end of the experiment.

*Group III: Drug Treated Control.* In this group, the animals were administered *Averrhoa carambola* extract (ACE) at a dose of 25 mg/kg/b.wt/animal/day for the entire experimental period.

*Group IV: ACE Treated Experimental.* The animals of this group were provided ACE at a dose of 25 mg/kg b.wt./day for 5 consecutive days. ACE source was withdrawn 48 hrs before the first administration of DENA. CCl_4_ was given after 2 weeks as 3 times a week for 24 weeks.

The following parameters were taken into account for the study:

1. Morphological:
  - *Body Weight.* The weights of the animals from each group were recorded at the beginning and at the termination of the experiments.
  - *Tumor Incidence.* It is the number of mice carrying at least 1 tumor expressed as percentage incidence.
  - *Tumor Yield.* It refers to the total number of tumors per group (number of tumors/total number of mice).
  - *Tumor Burden.* The average number of tumors per tumor bearing mouse (total number of tumors in all mice/total number of tumor bearing mice).
2. Biochemical. All the animals were autopsied after the end of experiment, that is, 24 weeks, and the whole liver was taken out from each mice. Biochemical analysis for the following parameters was performed in the liver.
  - *Lipid Peroxidation (LPO).* The level of LPO in liver was measured in terms of thiobarbituric acid reactive substances by the method of Ohkhawa et al. [22]. Briefly, thiobarbituric acid (0.8%), sodium dodecyl sulfate (0.1%), and acetic acid (20%) were added to 100 mL. of the tissue homogenate for 60 min. It was cooled and extracted with N-butanol-pyridine, and the optical density was recorded at 532 nm. The content of TBAS was expressed in nmol/mg.
  - *Glutathione (GSH).* The level of reduced GSH was estimated by the method of Moron et al. [23]. The GSH content in the liver was measured spectrophotometrically, using Ellman's reagent with 5,5′-dithiobis 2-nitrobenzoic acid (DTNB) as a coloring agent, according to the method of Beutler et al. [24]. The absorbance was recorded at 412 nm with levels expressed as nmol/mg of protein.
  - *Catalase (CAT).* The enzyme activity was assayed in the liver by the method of Aebi [25]. The content was estimated at 240 nm by monitoring the disappearance of H2O2.
  - *Superoxide Dismutase (SOD)*. The activity of this enzyme was measured by utilizing the method of S. Marklund and G. Marklund [26].
  - *Total Proteins.* The protein contents in liver were measured by the method of Lowry et al. [27]. The absorbance was recorded at 680 nm.

## 3. Results and Discussion

**Figure 1:**
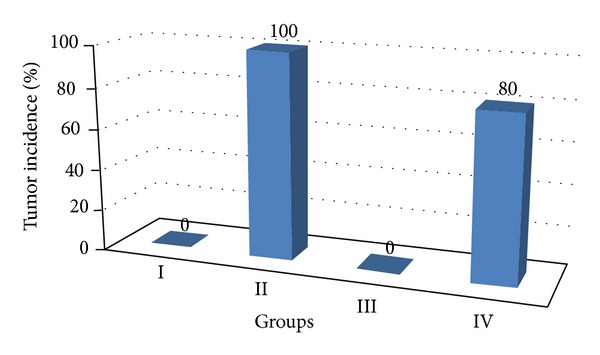
Variations in tumor incidence (%) after DENA/CCl_4_ induced hepatic carcinogenesis with/without ACE administration.

**Figure 2:**
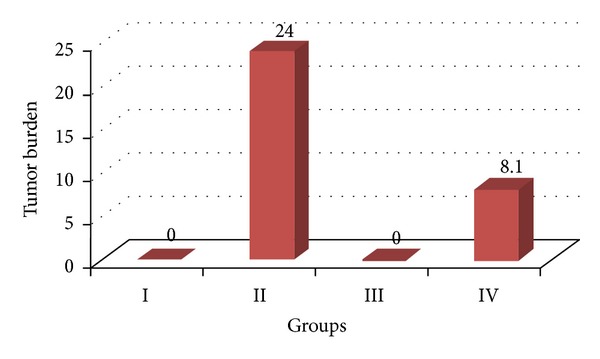
Variations in tumor burden in DENA/CCl_4_ induced hepatic carcinogenesis with/without ACE administration.

**Figure 3:**
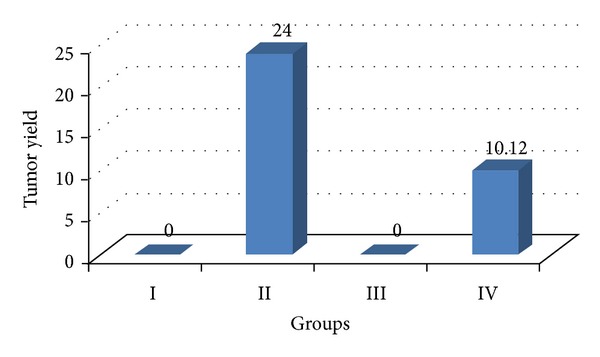
Variations in tumor yield after DENA/CCl_4_ induced hepatic carcinogenesis with/without ACE administration.

There was no considerable change in the average body weight of DENA (Group II), ACE (Group III), and DENA + ACE (Group IV) treated animals when compared to vehicle treated control (Group I). However, the ACE treated mice (Group III) exhibited a spontaneous gain in body weight as similar to the control mice. The mice receiving DENA and CCl_4_ (carcinogen control) exhibited a slight increase in mean body weight from that of the untreated (Group I) and ACE treated mice (Group III) till the end of experiment. On the contrary, liver weight was found to be significantly higher in DENA treated animals as compared to the ACE treated once. Further, no tumor appeared on the liver of the vehicle treated and ACE treated control animals while DENA treated mice had the appearance of the tumor incidence as 100%. On the other hand, such tumor appearance was reduced to 80% when DENA (Group III) treated mice were orally administered ACE (Group IV). Similarly, treatment with the carambola fruit extract leads to reduction in tumor burden and tumor yield to 33.75% and 42.16%, respectively, (Table 1, Figures 1, 2, and 3).

Morphologically several small white-grayish foci were detected on the liver of DENA treated mice at the end of the experimentation (i.e., 24 weeks). However, animals of vehicle treated control (Group I) as well as ACE treated control (Group III) did not show any such foci on the liver. On treatment of ACE with DENA (Group IV), the number of visible foci was found to be radically decreased and the liver surface was much smoother.

Induction of oxidative stress by DENA/CCl_4_ was evidenced in the liver by the increase in LPO level and fall in the activities of GSH, SOD, CAT, and total proteins content. The levels of LPO in liver were measured to be significantly raised (*P* < 0.001) to 12.91 ± 1.38 n mole/mg tissue, while the activities of GSH, SOD, and catalase as well as the level of total proteins in the liver were obtained to be significantly lower (*P* < 0.001) to 0.51 ± 0.02 *μ* mole/gm tissue, 12.59 ± 2.08 U/mg tissue, 2.06 ± 0.42 U/mg tissue, 33.31 ± 5.95 mg/gm, respectively, than the vehicle treated control values.

**Figure 4:**
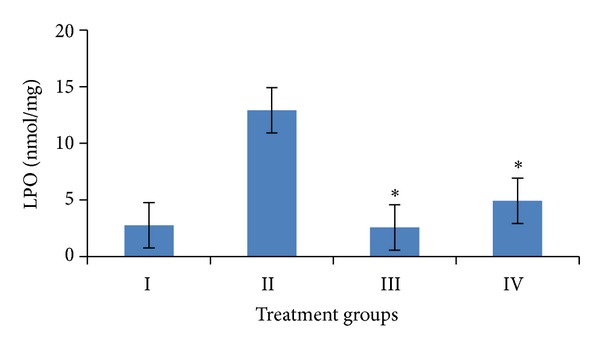
Variations in LPO levels after DENA/CCl_4_ induced hepatic carcinogenesis with/without ACE administration. Significance level—normal versus carcinogen treated control; carcinogen treated control versus ACE treated experimental *P < 0.001.

**Figure 5:**
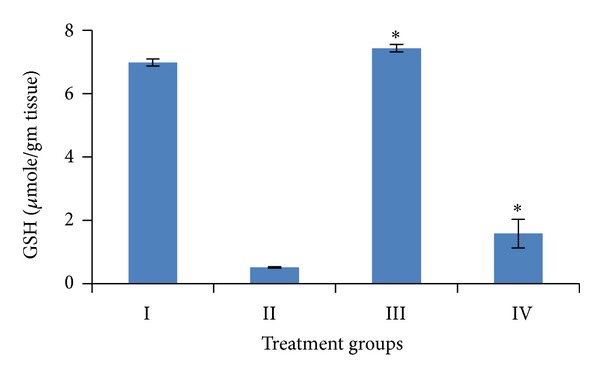
Variations in GSH level after DENA/CCl_4_ induced hepatic carcinogenesis with/without ACE administration. Significance level—normal versus carcinogen treated control; carcinogen treated control versus ACE treated experimental *P < 0.001.

**Figure 6:**
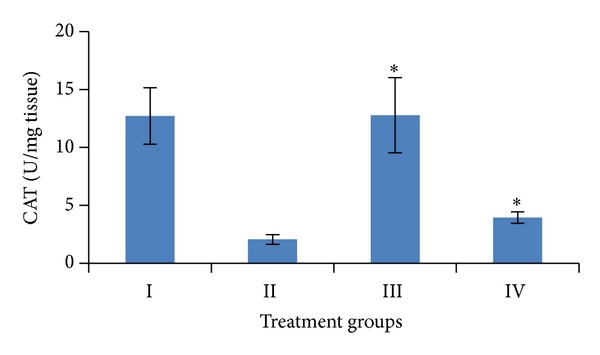
Variations in CAT level after DENA/CCl_4_ induced hepatic carcinogenesis with/without ACE administration. Significance level—normal versus carcinogen treated control; carcinogen treated control versus ACE treated experimental *P < 0.001.

**Figure 7:**
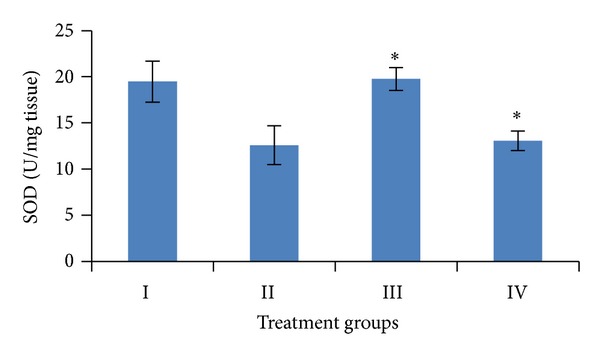
Variations in SOD level after DENA/CCl_4_ induced hepatic carcinogenesis with/without ACE administration. Significance level—normal versus carcinogen treated control; carcinogen treated control versus ACE treated experimental *P < 0.001.

**Figure 8:**
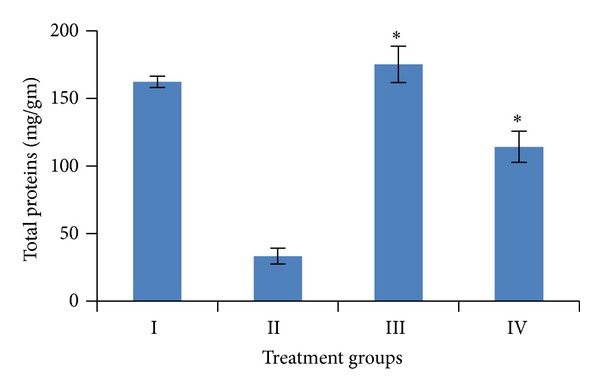
Variations in total proteins level after DENA/CCl_4_ induced hepatic carcinogenesis with/without ACE administration. Significance level—normal versus carcinogen treated control; carcinogen treated control versus ACE treated experimental *P < 0.001.

Pretreatment of mice with the *A. carambola *fruit extract (25 mg/kg b.wt. for 5 consecutive days and was withdrawn 48 hrs before the first administration of DENA) significantly (*P* < 0.001) lowered down the activity of LPO to 4.92 ± 0.89 n mole/mg tissue, GSH to 1.58 ± 0.45 *μ* mole/gm tissue, SOD to 13.05 ± 1.05 U/mg tissue, catalase to 3.95 ± 0.50 U/mg tissue, and the level of total proteins to 114.11 ± 11.55 mg/gm (Figures 4, 5, 6, 7, and 8).

Cancer continues to be a great challenge to scientists and practitioners interested in its biology, prevention, and therapy. Therefore, the search for new chemopreventive and antitumor agents, as more effective and less toxic than the existing ones, has kindled great interest in research for phytochemicals.

HCC is a complex disease with multiple underlying pathogenic mechanisms caused by a variety of risk factors. Hepatic carcinogenesis has been intensively studied in experimental animals, and numerous chemical compounds have been demonstrated to be carcinogenic to liver cells. DENA is used as hepatocarcinogen in this study, causing the tumor of the liver and not affecting any other organ. It has been used as an initiating agent in hepatocarcinogenic two-stage protocols, that is, initiation and promotion model.

There is a significant increase in the liver weight of mice receiving DENA and CCl_4_ as compared to vehicle treated control. It may be because of the presence of tumors and the increased size of liver in such animals. The increase in the size of liver might be because hypertrophy took place in the liver to compensate the damage induced by the carcinogens. After 24 weeks of DENA treatment, hyperplastic nodules developed as a consequence of the appearance of renewed hepatocytes, degenerated hepatocytes, oval cells, and fibrotic changes.

Marked elevations in biochemical parameters like LPO and the fall in GSH, SOD, catalase, and total protein levels in the liver reflect the degree of hepatocellular dysfunctions which indicates that reactive oxygen species, induced by DENA, play an important role in DENA-induced hepatic carcinogenesis. Therefore, it is suggested that oxidative stress is one of the major causes of DENA-induced hepatic carcinogenesis.

Oxidative stress is the state of imbalance between the level of antioxidant defense system and production of reactive oxygen species (ROS). Increased generation of ROS and decreased antioxidant enzymes in liver tissue has been reported in many models of DENA-induced hepatocellular carcinoma [28–31].

The implication of reactive oxygen species (ROS) in carcinogenic nitrosamines, like DENA and CCl_4_ toxic hepatic injury, is well documented [32]. It has been reported that ROS play a major role in tumor promotion through interaction with critical macromolecules including lipids, DNA, DNA repair systems, and other enzymes [33]. Increased O_2_ concentration and production of ROS, such as superoxide radical (*O_2_), hydroxyl radical (OH*), and hydrogen peroxide, cause oxidative stress in biological tissues. It may also act as tumor initiator by directly activating oncogenes through mutagenesis.

Herbal drugs play a role in the management of various liver disorders in addition to other natural healing processes of the liver [34]. There are studies which show that medicinal plants with hepatoprotective properties mediate their protection via antioxidant and free radical scavenging activities [35–37].

Further, the incidences of hepatic tumors were found to be significantly decreased in the experimental group (ACE treated) than the carcinogen treated control. The increase in the activities of the antioxidant enzymes in the experimental mice is attributed to the major antioxidative compounds present in the *Averrhoa carambola* fruit. These include catechin, epicatechin, proanthocyanidins, and saponins [38, 39]. Polyphenols and flavonoids are known to have hepatoprotective role [40, 41].

In the present study fruit extract of *Averrhoa carambola* prevented the progression of DENA-induced hepatic carcinogenesis. Data presented here demonstrated that administration of ACE reversed the decrease in GSH, CAT, SOD, and total proteins induced by DENA in liver tissues. Significant reduction in the LPO levels elicited by carambola and enhanced GSH, SOD, catalase, and proteins levels suggest the protection of structural integrity of hepatocytes cell membrane or stimulatory effects on hepatic regeneration, also reflecting the recovery of liver from the toxic effects of DENA and CCl_4_ towards the normal liver cell functions.

Lipid peroxidation plays an important role in the process of carcinogenesis [42] and may lead to the formation of several toxic products, such as malondialdehyde (MDE) and 4-hydroxynonenal. These products can attack cellular targets including DNA, thereby inducing mutagenicity and carcinogenicity [43]. Increase in lipid peroxidation has been reported during DENA-induced hepatocarcinogenesis [44]. An elevated level of lipid peroxidation during liver carcinogenesis was also observed in DENA treated control mice during the present study. Administration of carambola fruit extract resulted in a significant decrease in the levels of hepatic lipid peroxidation. The phytosterols present in the fruit of carambola showed to mediate the decrease in lipid peroxidation [45, 46].

Free radical scavenging enzymes such as superoxide dismutase (SOD) protect the biological systems from oxidative stress. SOD and CAT provide the first defense against oxygen toxicity by catalyzing the dismutation of superoxide anion to hydrogen peroxide and decomposition of hydrogen peroxide to water and molecular oxygen. Earlier reports showed the decreased activities of SOD and CAT in hepatoma [47]. The current study showed a significant decrease in SOD and CAT activity in mice treated with DENA. Decreased activities of SOD and CAT in DENA-treated mice could be due to overutilization of these nonenzymatic and enzymatic antioxidants to scavenge the products of lipid peroxidation. On the other hand, there was a significant increase in SOD and CAT activities in group treated with plant extract. It may be due to presence of the ascorbic acid which is known for its quenching abilities of the free radicals as well as for the conjugation with cytotoxic, genotoxic and lipid peroxidation products to ultimately lead their excretion [47, 48].

Glutathione is required to maintain the normal reduced state of cells and to counteract all the deleterious effects of oxidative stress. Thus, GSH is involved in many cellular processes including the detoxification of endogenous and exogenous compounds. The elevated level of GSH protects cellular proteins against oxidation through glutathione redox cycle and also directly detoxifies reactive species [49] while the increased level of glutathione reductase helps in maintaining the basal level of cellular GSH [50]. Administration of DENA depleted the level of glutathione (GSH) in this study. Such depletion is also reported in many studies [51–53]. It has been proposed that glutathione peroxidase is responsible for the detoxification of hydrogen peroxide in low concentration whereas catalase comes into play when glutathione peroxidase is saturated with the substrate [54]. GSH level was observed significantly higher in ACE treated mice than the carcinogen alone treated ones.

## 4. Conclusion

The exact mechanism of the chemopreventive action of ACE against DENA-induced hepatic tumor is not studied in the present experiment, but this investigation demonstrates that the *Averrhoa carambola* fruit extract has a prophylactic role against chemical induced hepatic carcinogenesis in the mammals.

## Figures and Tables

**Table 1:** Variations in body weight, liver weight, and morphometry of liver tumor after DENA/CCl_4_ treatment with or without ACE treatment.

Group	Body weight (gm)		Liver weight (gm)	Tumor		
	Initial	Final		Incidence (%)	Burden	Yield
I	13.10 ± 1.48	31.60 ± 0.37	2.54 ± 0.92	0	0	0
II	12.60 ± 0.34	35.00 ± 0.44	3.90 ± 0.54	100	24	24
III	15.40 ± 1.83	33.83 ± 0.97	2.60 ± 0.28	0	0	0
IV	12.50 ± 1.19	30.93 ± 0.22	3.05 ± 0.33	80	8.1	10.12
